# FDTransUnet: An aluminum surface defect segmentation model based on feature differentiation

**DOI:** 10.1371/journal.pone.0320060

**Published:** 2025-03-19

**Authors:** Mingzhu Tang, Wencheng Wang

**Affiliations:** 1 College of Mechanical and Control Engineering, Guilin University of Technology, Guilin, China; 2 Key Laboratory of Advanced Manufacturing and Automation Technology Guilin University of Technology, Education Department of Guangxi Zhuang Autonomous Region, Guilin, China; Shijiazhuang Tiedao University, CHINA

## Abstract

Aiming at the current problems in the field of industrial defect segmentation, such as difficulty of obtaining a large number of defect samples, low recognition accuracy and lack of segmentation accuracy, a surface defect segmentation model for aluminum based on feature differentiation is proposed: FDTransUnet. First, the limited defective samples are effectively expanded by the feature differentiation data augmentation strategy to alleviate the overfitting problem caused by the insufficient sample. Second, the Transformer architecture is added by improving the U-net network, and the improved network combines the global self-attention mechanism of the Transformer and the hierarchical structure of the U-net, which can effectively extract the local and global information in the defect sample. Finally, a composite loss function is constructed to address the problem of unbalanced foreground and background sizes of defective samples and to improve segmentation accuracy. The experimental results show that FDTransUnet achieves 94.5% MPA and 89.7% Dice coefficient on the aluminum surface defect dataset. In the final generalization experiment, FDTransUnet is validated with other mainstream segmentation models on the steel surface defect dataset, and the experiment proves that the segmentation model has good generalization performance and robustness, and can be applied to different scenarios of industrial inspection.

## Introduction

Aluminum is a material made from aluminum and its alloys. Aluminum is a light-weight, corrosion-resistant metal with good thermal conductivity and malleability, so it is widely used in a variety of industrial fields and in daily life. In the prospect of the rapid development of global manufacturing, aluminum, as an important metal material, is widely used in the fields of construction, automobile, aviation, electronics, and rail transportation. With the continuous upgrading of industrial industry, the requirements for the quality of aluminum are becoming more and more stringent. However, there are many problems in the traditional steel, aluminum and other metal manufacturing industries. Due to the defects in technology and production process, surface defects inevitably appear in the production of aluminum, which have a great impact on the aesthetics and quality of products. Therefore, the issue of quality inspection of the surface of aluminum has become particularly important.

At present, for this problem, many aluminum manufacturers still rely on manual visual inspection for aluminum surface defect detection. However, the manual visual inspection method not only requires a large amount of manpower, but also exists such as a high rate of misjudgment, strong personal subjective factors, inefficiency and other problems. In addition to manual visual inspection methods, there are manufacturers using machine vision methods to assist in the inspection of product quality.

Recent years have brought significant breakthroughs and advances in the field of image segmentation by the development of computer vision and researchers’ focused studies. In 2014, Jonathan Long et al. [1] proposed the Fully Convolutional Network (FCN). FCN proposed to replace the fully connected layer with a convolutional layer, so as to realize the pixel-level classification of the image, that is, each pixel is classified, thus solving the problem of image segmentation at the semantic level. However, the segmentation results are not fine enough, sensitive to details, and do not consider the relationship between pixels, and lack of spatial consistency. In 2015, Olaf Ronneberger et al. [2] proposed the U-net network for the medical image segmentation problem. U-net network utilizes a U-shaped encoder and decoder architecture and introduces skip connection. The skip connection connects the feature maps in the encoder to the corresponding layers in the decoder, which helps to preserve detail information and mitigate the gradient vanishing problem. Jiwei Lu et al. [3] attempted to apply deep learning algorithms to the surface defect segmentation of steel, and proposed a defect segmentation method based on Gabor filter image fusion and discrete Fourier spectrum residuals. This method realizes the effective segmentation of defects, and to some extent can be used as a reference method for the segmentation of steel defects. Dongyu Lin et al. [4] to address the problem of defect detection and segmentation of industrial images, they proposed an improved U-net network that can be trained on a small number of defective samples with only labeling information, which effectively alleviates the problem of few defective samples. Chuxin Yang et al. [5] proposed a background reconstruction method based on cycle generative adversarial network to solve the problem of difficulty in labeling defect samples, and then used U-net network as the segmentation model. The experimental results show that it can obtain good defect segmentation results with a small number of defect samples.

To summarize, there are still many deficiencies in the current direction of metal surface defect segmentation based on deep learning, such as few and difficult to obtain defect samples, large differences in defect category features, imbalance between foreground and background, and insufficient accuracy of segmentation [6]. The existence of these problems brings great challenges to the defect segmentation task. To address the above problems, this paper proposes an aluminum surface defect segmentation model based on feature differentiation, which is contributed in this paper as follows:

1. A data augmentation strategy based on feature differentiation is proposed, which simulates the defect samples, not only simply replicates the defect, but deliberately differentiates the simulated defect samples from the real defect samples. This strategy can not only improve the learning ability of the network, but also alleviate the overfit-ting problem caused by insufficient defect samples.
2. The TransUnet model is introduced as a segmentation network, and explores the potential of the Transformer architecture and the U-net network in the field of industrial defect segmentation. TransUnet model establishes a self-attention mechanism from a sequence-to-sequence prediction perspective, combining the respective advantages of the U-net and the Transformer.
3. To compensate for the loss of feature resolution problem associated with the Transformer architecture, a hybrid CNN-Transformer structure is used, which utilizes both the properties of CNN to obtain more detailed spatial information at high resolution and the global encoded by the Transformer.
4. Aiming at the problem of imbalance between foreground and background in aluminum defective samples, a composite loss function is redesigned, which takes into account the relationship between pixels and balances the results of the loss function, thus further improving the model performance.

## Materials

The dataset in this paper comes from the aluminum dataset of Aliyun Tianchi Competition, which is a public dataset. The dataset samples are collected from the actual production of Foshan Nanhai aluminum manufacturers with defective aluminum samples, using an industrial camera, the sample size of 1024 *  1024. The dataset contains six categories of defects, respectively, Missing paint, Surface bulge, Surface impurity, Pit, Wrinkle, and Coating crack.

The collected aluminum defect samples contain six categories with a total of 360 images. The defect samples were increased to 1080 sheets after feature-differentiated data augmentation, and the dataset was divided into training set samples and validation set samples and test set samples in the ratio of 7:2:1. The specific number of categories is shown in Table 1.

**Table 1:** Aluminum defect dataset.

Category	Augmentation of defective samples	Training	Validation	Test
Missing paint	294	206	58	30
Surface bulge	153	107	31	15
Surface impurity	99	70	19	10
Pit	162	114	32	16
Wrinkle	222	156	44	22
Coating crack	150	105	30	15

## Method

**Fig 1:**
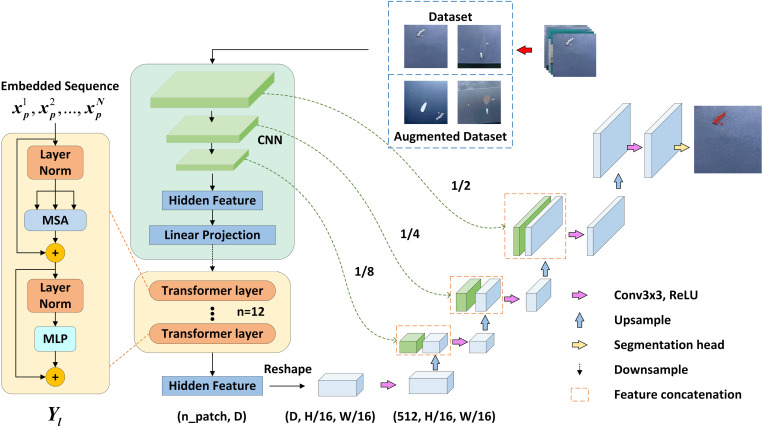
FDTransUnet network structure diagram.

This section will introduce the proposed aluminum surface defect segmentation model: FDTransUnet. It will be illustrated by three aspects: feature differentiation data augmentation strategy, TransUnet segmentation network, and composite loss function, and its network structure is shown in Fig 1.

### Feature differentiation data augmentation strategy

**Fig 2:**
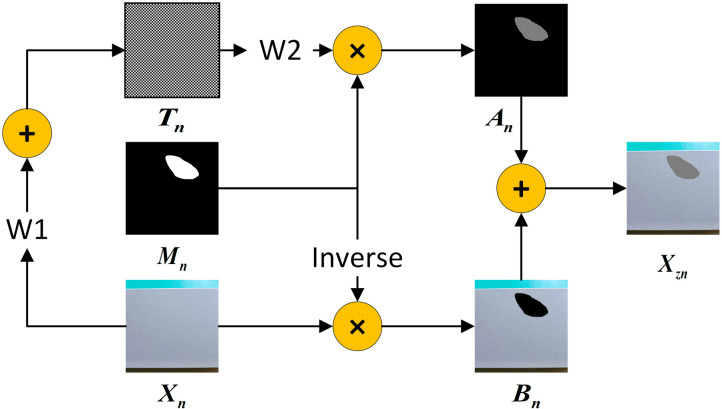
Feature differentiation data augmentation structural diagram.

In the actual production of aluminum defective samples are difficult to collect and difficult to obtain, but normal samples are relatively simple to obtain. Therefore, this paper proposes a feature differentiation data augmentation strategy. First use the normal aluminum sample obtained denoted as ${\text{X}}_{\text{n}}$ . secondly using a small number of anomalous blocks, Gaussian noise, regular lattice and similar dataset defective anomalous blocks to produce different types of noise blocks ${\text{T}}_{\text{n}}$. Finally, a mask block ${\text{M}}_{\text{n}}$ is synthesized from the Bessel random curve [7] and the labeled defects. The data augmentation strategy process is shown in Fig 2.

Under the condition of limited defect samples, the use of data augmentation strategy can effectively enhance the utilization of defective samples and also greatly alleviate the problem of insufficient dataset. Where ${\text{A}}_{\text{n}}$ is the target region defective splicing block and ${\text{B}}_{\text{n}}$ is the background region block, which is calculated as follows:


$$A_{n}=\left(W_{1}X_{n}⊕W_{2}T_{n}\right)⊗M_{n}$$ (1)

$$B_{n}=X_{n}⊗Inv\left(M_{n}\right)$$ (2)

where W1 and W2 are the transparency weights of the background block and the noise block respectively, which are each set to 0.5 in this paper. After taking the inverse, the defective region will be changed to black and the background region will be changed to white. After obtaining the defect splicing block ${\text{A}}_{\text{n}}$ and the background block ${\text{B}}_{\text{n}}$, combine them together to obtain the final augmented image ${\text{X}}_{\text{zn}}$, the formula is as follows:


$$X_{zn}=A_{n}⊕B_{n}$$ (3)

**Fig 3:**
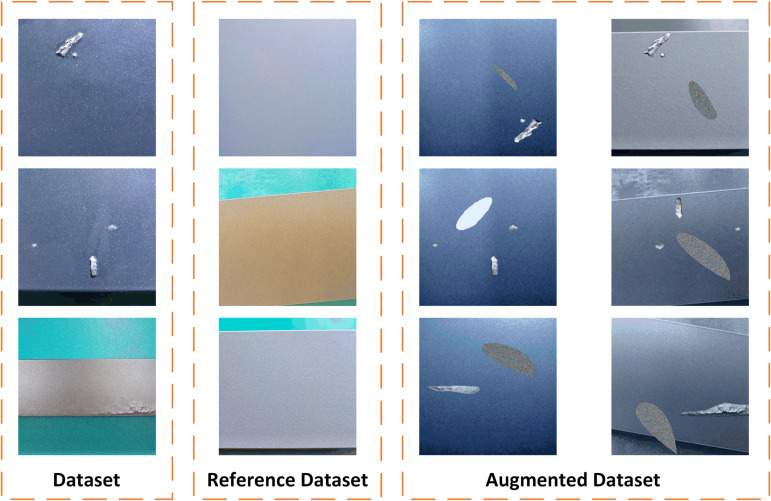
Sample of partial augmentation defects in aluminum.

A partial sample of the data augmentation is shown in Fig 3. After the above data augmentation strategy, a large number of simulated aluminum defect samples can be obtained, which contains the defect structure and texture, and the simulated defect range and defect size are similar to the real defects, but his defect shape and defect content are different from the real defects, which makes the simulated defect samples and real defect samples similarity has been improved, and the difference has been improved.

### TransUnet segmentation network

U-net is an image segmentation network widely used in the field of computer vision [8]. U-net is known for its U-shaped network structure. This network model was originally designed to solve problems in medical image segmentation, such as cell segmentation and organ segmentation. U-net it cleverly integrates the encoder and decoder, and realizes the effective capture of multi-scale semantic information in images through the skip connection mechanism. This integration not only enhances the network’s ability to perceive image details, but also significantly improves the distinction between foreground and background. The skip connection mechanism can transfer the features captured in the encoding stage directly to the decoding stage, which can effectively mitigate the gradient vanishing problem in the deep network training, and at the same time reduce information loss and ensure the integrity of feature information.

TransUnet [9] segmentation network integrates the Transformer [10] architecture perfectly into U-net, which retains the advantages of Transformer for obtaining global sequence information and the U-net network for extracting more image details in sample processing. In this paper, TransUnet is used as the basic segmentation network architecture, which utilizes both the U-net’s function of extracting data features and the Transformer’s role of encoding global feature locations, thus giving TransUnet the capability of obtaining global information about the data.

**Fig 4:**
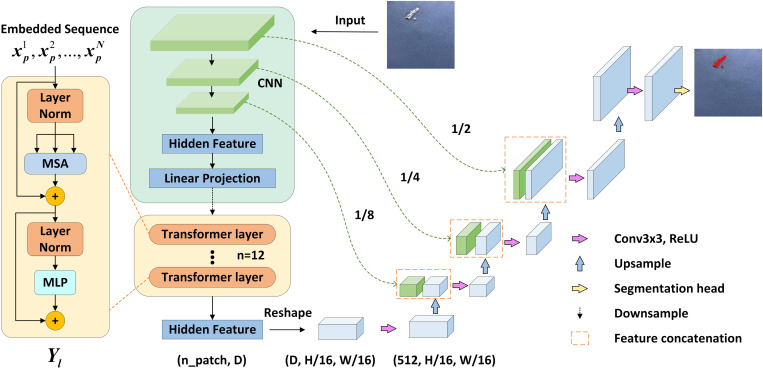
TransUnet network structure diagram.

The network framework of TransUnet consists of three important modules: CNN-Transformer encoder, decoder, and skip connection. Transformer’s use of the self-attention mechanism for global feature extraction may tend to focus on positional information, leading to the neglect of many aspects of local information [11]. In contrast, U-net excels at local feature extraction but lacks the ability to comprehensively acquire detailed information. The structure of TransUnet is shown in Fig 4.

If CNN is used as an encoder alone, although CNN has better feature extraction and perceptual ability, and different sizes of convolutional cores have different receptive fields, the disadvantage is that it cannot synthesize global information, and it will lose part of the feature information when pooling operation is carried out, and lacks the correlation between the local and the whole. And Transformer is just the opposite, because it has a self-attention mechanism, it will focus to the feature extraction of global information, but the disadvantage is that the ability to extract detailed information is weak.

In this paper, the encoder with CNN-Transformer architecture is used, which employs the properties of CNN to obtain more detailed spatial information at high resolution, and then improves the inability of convolutional networks to establish long-distance dependencies through the Transformer’s unique self-attention mechanism.

TransUnet first inputs the defective samples into the CNN for information extraction, and then performs 3 downsampling operations in the CNN to reduce the size of the feature map to 1/2, 1/4, and 1/8 of the input sample. After the sampling is completed, the sampled sample is processed, firstly, the sample is divided into a bunch of patches, and then divided according to the given size, each patch is mapped to a one-dimensional vector by linear mapping and outputs a sequence of vectors, and then finally inputs into the Transformer layer and loops 12 times.

The Transformer layer with global self-attention is based on the Vision Transformer (ViT) [12], which contains three modules: Layer norm, Multi-head Self-attention (MSA), and Multi-layer Perceptron (MLP) [13]. The output of MSA and MLP in each Transformer layer can be expressed by the following formula:


$${Y^{\prime }}_{l}=MSA\left(LN\left(Y_{l-1}\right)\right)+Y_{l-1}$$ (4)

$$Y_{l}=MLP\left(LN\left({Y^{\prime }}_{l-1}\right)\right)+{Y^{\prime }}_{l-1}$$ (5)

where ${\text{Y}}_{\text{l}-1}$ is the output of the layer l−1 Transformer and ${\text{Y}}_{\text{l}}^{\text{'}}$ is the residual connection between the output after MSA and ${\text{Y}}_{\text{l}-1}$. ${\text{Y}}_{\text{l}}$ is the output of the layer l Transformer, which is obtained by concatenating the output of the MLP with the $Y_{l}^{\text{'}}$ residual and is used as the input of the next Transformer layer. After the Transformer encoder extracts the global deep features of the defects, the decoder is constructed by utilizing the same convolution as well as up-sampling operations as in U-net. Thus, the dimension of the feature map and sequence features is increased, and the final prediction result is restored to the original input size.

### Composite loss function

When the network model is built, the loss function serves to measure the gap between the model predictions and the labeled values when training the model. The loss function used in the training process of TransUnet is Dice loss [14], Dice loss is a loss function commonly used in segmentation models, the basic idea is to improve the accuracy by calculating the overlap between the predicted results and the real results and reducing the difference between them. The formula of Dice loss is as follows:


$$DiceLoss=1-\frac{2\sum_{i=1}^{N}p_{i}g_{i}}{\sum_{i=1}^{N}p_{i}^{2}+\sum_{i=1}^{N}g_{i}{}^{2}}$$ (6)

where $p_{i}$ is the pixel value of the real sample and $g_{i}$ is the pixel value of the predicted sample. *N* is the number of categories of defective labels.

From the above formula, it can be observed that when using Dice loss, severe oscillations may occur when the aluminum defect sample is a small target. This is because in the case where there is only foreground and background in the sample and the target in the foreground is small, an incorrect prediction of the small target can cause a large fluctuation in the loss function, which can lead to a drastic change in the gradient along with it. And when the foreground and background are not balanced, Dice loss will easily overfitting. Considering the above problems and the fact that only the unbalance between foreground and background is very common in aluminum defective samples, this paper introduces cross-entropy (CE) loss [15] function on the basis of Dice loss to construct a composite loss function, which is calculated by the following formula:


$$CELoss=-g_{i}log(p_{i})-\left(1-g_{i}\right)log(1-p_{i})$$ (7)

CompositeLoss=α(DiceLoss)+β(CELoss) (8)

Here α and β are the hyperparameters of the training loss. Composite loss function is constructed by combining Dice loss and CE loss. Composite loss function reconsiders the relationship between pixels and restricts the estimation of the result loss of the model, so as to more accurately segment aluminum defects.

## Experiment and result analysis

In this section, we will detail the specific environment and parameter settings used for the experiments, as well as the metrics used for evaluation. We will also provide a detailed experimental protocol to ensure the repeatability and verifiability of the experiments. Finally, we will provide a comprehensive analysis of the experimental results to fully demonstrate the validity of the experiments and show the significance of the model improvements.

### Experimental environment and parameters

The environment for the experiments in this paper: Intel Core i5-12400F processor, NVIDIA GeForce RTX 2060Super graphics card, Python version 3.9.13, the PyTorch framework, and the OpenCV image processing tool. The specific training parameters are shown in Table 2.

**Table 2:** Training parameters.

Learning rate	Optimizer	Momentum	Batch size	Weight decay	Epoch
0.01	SGD	0.9	8	0.0001	50

### Evaluation metrics

In order to observe the performance of the model more intuitively, in this paper, the typical segmentation model evaluation metrics are used, such as precision (P), pixel accuracy (PA), category average pixel accuracy (MPA), Dices score and mean intersection over union (MIoU) [16]. The formula is as follows:


$$P=\sum_{i=0}^{k}\frac{p_{ii}}{\sum_{j=0}^{k}p_{ij}+p_{ii}}$$ (9)

$$PA=\frac{\sum_{i=0}^{k}p_{ii}}{\sum_{i=0}^{k}\sum_{j=0}^{k}p_{ij}}$$ (10)

$$MPA=\frac{1}{k+1}\sum_{i=0}^{k}\frac{p_{ii}}{\sum_{j=0}^{k}p_{ij}}$$ (11)

$$MIoU=\frac{1}{N+1}\sum_{i}^{N}\frac{TP}{FN+FP+TP}$$ (12)

$$Dice=\frac{2TP}{2TP+FP+FN}$$ (13)

where: *k* is the total number of pixels in the image, $p_{ii}$ is the total number of pixels in class *i* predicted to be in class *i*, $p_{jj}$ is the total number of pixels in class *j* predicted to be in class *j*, $p_{ij}$ is the total number of pixels that would have belonged to class *i* predicted to be in class *j*, and $p_{ji}$ is the total number of pixels that would have belonged to class *j* predicted to be in class *i*.

### Loss function experiments

In order to verify the effectiveness of the Composite loss function, Dice loss, CE loss and Focal loss [17], which are commonly used in segmentation tasks, are selected for comparison experiments with the composite loss function proposed in this paper. Using TransUnet as the segmentation network, the results of the loss function comparison experiments are shown in Table 3 below.

**Table 3:** Loss function comparison experiments.

Model	MPA/%	MIoU/%	Dice/%
TransUnet + Dice loss	85.2	72.0	82.2
TransUnet + CE loss	83.6	70.7	81.5
TransUnet + Focal loss	86.9	74.2	83.4
TransUnet + Composite loss	88.7	76.7	85.6

From the loss function comparison experimental results table, it can be seen that using Dice loss or CE loss alone is not very effective in the aluminum defect segmentation task, and Focal loss is only slightly better. Composite loss function outperforms the other loss functions in MPA, MIoU and Dice score, and has good results in aluminum defect segmentation.

**Fig 5:**
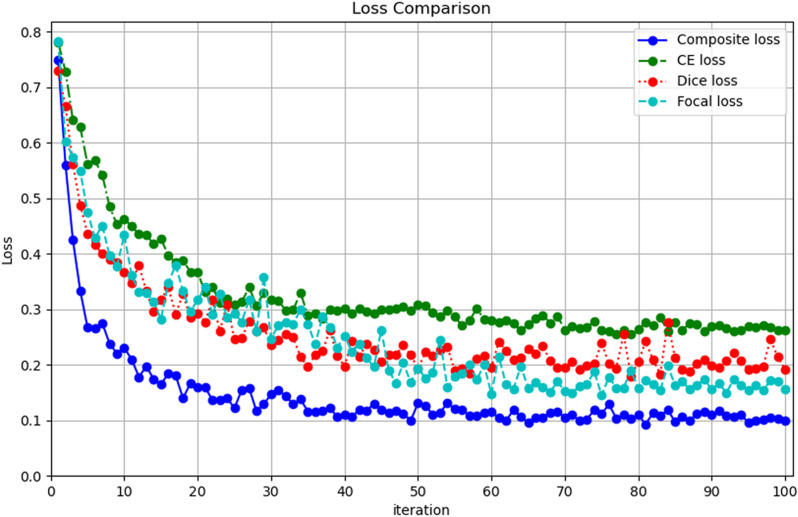
Loss function comparison curve.

During the training process of the loss function comparison experiment, the training loss of the model is recorded after the completion of each iteration, and the first iteration at the beginning of each epoch as well as the intermediate iteration are recorded and plotted as loss curves, as shown in Fig 5. The vertical coordinate is the value of the loss and the horizontal coordinate is the iteration, with 50 training epochs corresponding to 100 iterations. From Fig 5. It can be observed that the composite loss decreases and converges faster and has the best results. Dice loss, CE loss and Focal loss are inferior to the Composite loss, and there are large fluctuations in the decreasing loss curves of Dice loss and Focal loss.

It was mentioned in the previous section that there are two hyperparameters in Composite loss function: α and β, whose value intervals are 0-1. The following table shows the ablation experiments when different values of α and β are selected. From Table 4, it is observed that the optimal segmentation results are achieved when α is set to 0.6 and β is set to 0.4.

**Table 4:** Loss function hyperparametric ablation experiments.

*α*	*β*	MIoU/%	Dice/%
0.2	0.8	72.3	82.3
0.4	0.6	74.5	83.6
0.5	0.5	75.4	84.4
0.6	0.4	76.7	85.6
0.8	0.2	73.9	83.3

### Ablation experiment

In order to verify the effectiveness of the FDTransUnet segmentation model proposed in this paper, the experiment is divided into eight parts for ablation experiments, and here the U-Net model is used as the baseline model, and the results of the ablation experiments are shown in Table 5.

**Table 5:** Ablation experiments.

Sequence	Model	MPA/%	MIoU/%	Dice/%
1	U-net	78.9	68.9	76.3
2	U-net + FD	81.4	70.8	79.7
3	U-net + Composite loss	80.3	70.1	78.2
4	U-net + FD + Composite loss	84.0	72.3	81.8
5	TransUnet	85.2	73.0	82.2
6	TransUnet + FD	90.4	76.8	87.1
7	TransUnet + Composite loss	88.7	76.7	85.6
8	TransUnet + FD + Composite loss (Ours)	94.5	81.3	89.7

In the above table FD is Feature differentiation data augmentation strategy. The experimental data show that when using U-net as the baseline segmentation network, adding data augmentation and Composite loss alone can improve MIoU by 1.9% and 1.2%, and Dice by 3.4% and 1.9%. It shows that both data augmentation and Composite loss function have better results on different segmentation models. When U-net added the two improvements MIoU and dice improved by 3.4% and 5.5%, respectively.

When the baseline segmentation model is switched to TransUnet, the advantages of TransUnet, which combines the respective advantages of Transformer and U-net, can be observed, with accuracy and model precision far exceeding that of U-net. When it added data augmentation and Composite loss, MIoU and dice came to 81.3% and 89.7%, respectively, which were 8.3% and 7.5% higher compared to before addition. Finally, the FDTransUnet segmentation model proposed in this paper improves 15.6%, 12.4% and 13.4% compared to the U-net model MPA, MIoU and dice respectively. This shows that the FDTransUnet segmentation model achieves good results above the aluminum defect segmentation task.

### Model comparison experiment

To verify the comprehensive performance of the improved model in this paper, we conducted comparison experiments using the model proposed in this paper with other current mainstream segmentation models. In the experiments, FDTransUnet is compared with five models including U-net, U-net++ [18], Deeplabv3+ [19], PSPnet [20] and TransUnet. And the same experimental parameters, configurations and datasets are used, and finally the results of the comparison experiments are compared and analyzed by the four evaluation metrics of P, MPA, MIoU and Dice score, and the experimental results of the precision of the comparison models are shown in Table 6 below.

**Table 6:** Comparison of model precision experiments.

Title	U-net	U-net++	Deeplabv3+	PSPnet	TransUnet	FDTransUnet (Ours)
Missing paint	78.7	79.4	84.8	80.6	87.5	92.8
Surface bulge	81.3	84.0	87.9	82.3	90.3	94.1
Surface impurity	83.9	85.6	88.5	77.9	91.9	97.2
Pit	76.1	79.7	82.7	78.1	88.8	94.5
Wrinkle	77.3	81.6	83.1	78.2	86.1	85.9
Coating crack	79.2	83.5	86.9	77.2	86.0	87.2

It can be seen that the segmentation accuracy of U-net and PSPnet is less effective, and the gap with other segmentation models is large. Our proposed model outperforms the other models in terms of precision for most of the categories, but the segmentation precision for the category wrinkle is slightly worse than that of TransUnet. It can also be seen in the table that the effect of the we model is significant for defects with small shapes and more obvious features such as missing paint, surface bulge, surface impurity and pit. However, the segmentation effect is not satisfactory when encountering large defects such as wrinkle and coating crack. Feature differentiation data augmentation strategy in the face of this large-area defects enhancement effect is not good, and wrinkle and coating crack of the two defects of the surface characteristics of the similar, these two reasons together so that the model of the two defects category segmentation precision is not much. The results of the specific model comparison experiments are shown in Table 7.

**Table 7:** Model comparison experiments.

Model	MPA/%	MIoU/%	Dice/%
U-net	78.9	68.9	76.3
U-net++	81.3	71.4	79.1
Deeplabv3+	84.4	73.0	81.5
PSPnet	77.2	65.1	73.9
TransUnet	85.2	73.0	82.2
FDTransUnet (Ours)	94.5	81.3	89.7

From the model comparison experiments, it is observed that from the evaluation metrics, FDTransUnet model proposed in this paper is the optimal segmentation effect in both MPA, MIoU and Dice, and the indexes are higher than the other comparison models. The MIoU value of FDTransUnet is 81.3%, which is 12.4%, 9.9%, 8.3%, 16.2%, and 8.3% higher as compared to the other models, indicating that the segmentation region is more appropriate. The MPA value of FDTransUnet is 94.5%, which is 15.6%, 13.2%, 10.1%, 17.3%, and 9.3% higher compared to the other models, indicating that the segmentation target is more accurate. The above results show that the model proposed in this paper has better segmentation accuracy in the aluminum surface defect segmentation task and is more suitable for the aluminum surface defect segmentation task.

**Fig 6:**
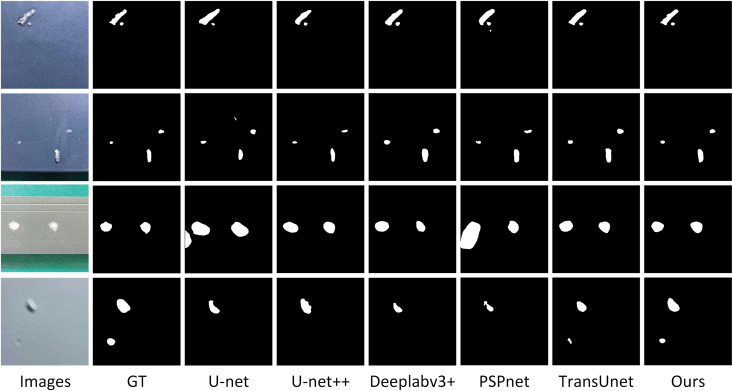
Visualization of model comparison experiments diagram.

Fig 6 shows a comparison of the visualized model segmentation, where GT is the real label, and from left to right are U-net, U-net++, Deeplabv3+ , PSPnet, TransUnet and FDTransUnet. As can be observed from the figure, there are cases of misdetection and omission in U-net. U-net++ reduces misdetection and omission, but the segmentation effect is still poor. PSPnet segments very roughly and poorly. Deeplabv3+ and TransUnet segmentation effect is not bad overall, but performs poorly when the defective features are not obvious and when the target is small. FDTransUnet segments the details most accurately, without misdetection and omission, and fits the defects very closely. In summary, FDTransUnet is more applicable in the direction of aluminum surface defect segmentation compared to other mainstream models.

### Generalization experiment

In order to verify that the proposed model is robust and can be applied to different industrial scenarios, the proposed model in this paper is generalized and compared with the mainstream segmentation models mentioned in the previous section on the RSDDs (rail surface discrete defect) public dataset [21]. The RSDDs dataset was collected from the railroad lines that were put into use, and the collected steel images were divided into defective samples and normal samples, which contained a total of 600 samples, and the size of the samples was 256 * 256. The experiments in this section will also use the same experimental parameters and configurations as in the previous section, the dataset will use the RSDDs dataset, and the evaluation metrics will be analyzed using IoU and Dice score, and the results of the generalization experiments are shown in Table 8.

**Table 8:** Generalization experiments.

Model	MIoU/%	Dice/%
U-net	72.4	78.1
U-net++	77.1	83.8
Deeplabv3+	79.4	84.1
PSPnet	70.3	77.6
TransUnet	82.0	88.1
FDTransUnet (Ours)	89.8	95.4

According to the generalization experiments in Table 8, FDTransUnet still maintains the lead in accuracy in steel surface defect segmentation, with IoU reaching 89.8% and Dice reaching 95.4%.

**Fig 7:**
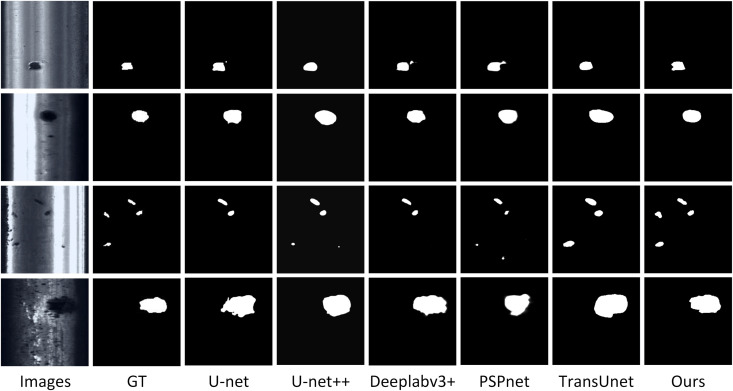
Visualization of generalization experiments diagram.

Observing the visualization of the generalization experiment in Fig 7, it can be found that except for FDTransUnet, the other mainstream segmentation models have more or less missed and misdetection when facing small targets. The model in this paper still has the advantage of segmentation accuracy over other mainstream models on the steel defect segmentation dataset, which verifies that the model has excellent generalization performance and robustness, and can be applied to different scenarios of industrial segmentation.

## Discussion

After Transformer was applied to the field of computer vision, it challenged the dominance of convolutional neural networks in the field of computer vision with extremely high performance, realizing a great unification between the field of natural language processing and the field of computer vision. Since then, there have been numerous combinations of variants of Transformer with other networks. The biggest problem of Transformer is that the computational cost is extremely high, which makes it difficult to be applied to small volumes of industrial defective image data. And one of the characteristics of U-net network is that it requires small training set, simple network structure and less computational cost, which is very suitable for industrial defect image segmentation. In this context, although the proposed FDTransUnet model in this paper has good segmentation results in the aluminum defect segmentation task, the segmentation model still has significant limitations, such as the model is too bloated, the number of parameters, and the model is too large for mobile deployment.

For the model is too bloated, the number of parameters is too large, the size of the model can’t meet the requirements of mobile deployment, as well as real-time can’t be guaranteed and so on, the mainstream is generally used in the following two methods:

1. Compression of the model. Firstly, model pruning can be used to remove redundant parameters and unimportant connections, allowing the amount of model parameters to be reduced, and also trying to ensure that model accuracy is not compromised. Secondly, model quantization can be used to reduce storage and computational costs by converting model parameters to low-precision representations. Finally, there is knowledge distillation, which transfers knowledge from large models to small models to achieve model compression.
2. Use lightweight model. Adopting a model that is more lightweight than U-net, or by reasonably choosing the model structure, optimizing the design and deployment strategy, an efficient lightweight model can be achieved to meet the real-time requirements and obtain good performance on the mobile side.

To summarize, the actual deployment in industrial production is a major research direction, and is also our future research focus. Realize the deployment in actual production scenarios, and finally complete the integration and automation of the production inspection process.

## Conclusion

In this paper, for the industrial production of aluminum defect samples are difficult to obtain, aluminum defect sample category surface feature differences, the segmentation effect is not accurate enough and other issues, proposed a segmentation model for aluminum surface defects based on feature differentiation: FDTransUnet. First, a feature differentiation data augmentation strategy is used to solve the problem of difficult access to defect samples and alleviate the overfitting problem caused by too small a sample size. Then, the U-net is fused with the Transformer architecture, and TransUnet is utilized as the segmentation network for the aluminum defect segmentation task. Finally, a composite loss function is constructed to solve the problem of imbalance between the foreground and background of defective samples and improve the segmentation accuracy. Through a large number of experiments, it is proved that the FDTransUnet proposed in this paper has better segmentation effect in aluminum defect segmentation task, higher segmentation accuracy, stronger generalization ability, and more suitable for aluminum defect segmentation task.
